# Efficient Double Suzuki Cross-Coupling Reactions of 2,5-Dibromo-3-hexylthiophene: Anti-Tumor, Haemolytic, Anti-Thrombolytic and Biofilm Inhibition Studies

**DOI:** 10.3390/molecules21080977

**Published:** 2016-07-27

**Authors:** Hafiz Mansoor Ikram, Nasir Rasool, Muhammad Zubair, Khalid Mohammed Khan, Ghayoor Abbas Chotana, Muhammad Nadeem Akhtar, Nadiah Abu, Noorjahan Banu Alitheen, Abdallah Mohamed Elgorban, Usman Ali Rana

**Affiliations:** 1 Department of Chemistry, Government College University Faisalabad, Faisalabad 38000, Pakistan; chemistue@gmail.com (H.M.I.); nr_308@hotmail.com (M.Z.); 2 International Center for Chemical and Biological Sciences, HEJ Research Institute of Chemistry, University of Karachi, Karachi 75270, Pakistan; Khalid.khan@iccs.edu; 3 Department of Chemistry, SBA School of Science & Engineering, Lahore University of Management Sciences, Sector U, DHA, Lahore Cantt. 54792, Pakistan; ghayoor.abbas@lums.edu.pk; 4 Faculty of Industrial Sciences & Technology, University Malaysia Pahang, Lebuhraya Tun Razak 26300, Kuantan Pahang, Malaysia; nadeemupm@gmail.com; 5 Faculty of Biotechnology and Biomolecular Sciences, University Putra Malaysia, Serdang, Selangor Darul Ehsan 43400, Malaysia; nadyaboo@gmail.com; 6 Botany and Microbiology Department, College of Science, King Saud University, Riyadh 11451, Saudi Arabia; elgorban@yahoo.com; 7 Sustainable Energy Technologies (SET) Center, College of Engineering, King Saud University, Riyadh 11421, Saudi Arabia; urana@ksu.edu.sa

**Keywords:** Suzuki cross-coupling reaction, 2,5-biaryl-3-hexylthiophene derivatives, anti-tumor, biofilm inhibition, haemolysis, anti-thrombolytic

## Abstract

The present study describes several novel 2,5-biaryl-3-hexylthiophene derivatives (**3a**–**i**) synthesized via a Pd(0)-catalyzed Suzuki cross-coupling reaction in moderate to good yields. The novel compounds were also analyzed for their anti-thrombolytic, haemolytic, and biofilm inhibition activities. In addition, the anti-tumor activity was also evaluated in vitro for newly-synthesized compounds, where 3-hexyl-2,5-bis(4-(methylthio)phenyl)thiophene exhibited the best anti-tumor activity against 4T1 cells with IC_50_ value of 16 μM. Moreover, 2,5-bis(4-methylphenyl)-3-hexylthiophene showed the highest activity against MCF-7 cells with an IC_50_ value of 26.2 μM. On the other hand, the compound 2,5-bis(4-chloropheny)-3-hexylthiophene exhibited excellent biofilm inhibition activity. Furthermore, the compound 2,5-bis(3-chloro-4-fluorophenyl)-3-hexylthiophene also exhibited better anti-thrombolytic and hemolytic activity results as compared to the other newly-synthesized compounds.

## 1. Introduction

The Suzuki-Miyaura cross-coupling reaction is an important type of Pd-catalyzed coupling reaction, which involves the synthesis of biaryls by the reaction between aryl halides and organoboronic acids [1,2]. This typical coupling reaction is very useful and selective for carbon-carbon bond formation [3,4]. The specific advantages associated with the Suzuki-Miyaura cross-coupling reaction include ordinary reaction conditions, tolerance for a broad range of functional groups and non-hazardous byproducts [5]. Sulfur-containing substituted thiophene based compounds display great chemical and pharmaceutically important characteristics, including anti-inflammatory [6], anti-depressant [7], anti-HIV PR inhibitors [8], BACE1 inhibitors [9], anti-thrombolytic [10], anti-tumor [11] and anti-tubercular activities [12]. In addition, thiophene derivatives have shown successful applications for azo dyes [13], electrochromic devices [14], energy storage devices [15], non-linear optics [16], biodiagnostics, conductivity-based sensors, super conductors and optoelectronics [13]. Hence, the synthesis of new thiophene derivatives with the desired medicinal characteristics is receiving significant attention these days, in particular for the development of new drugs.

Previously, we have reported the synthesis of some novel thiophene derivatives, which displayed facile biological activities [10]. Hence, following our previous work and in the search of new therapeutic agents, herein, we describe the synthesis of various functionalized 2,5-biaryl-3-hexylthiophene derivatives using several organoboronic acids via Pd-catalyzed Suzuki coupling reactions. More importantly, we have investigated the biological activities of these important and novel thiophene based compounds against various diseases, where the results revealed good biofilm inhibition, haemolytic, anti-thrombolytic, and anti-tumor activities, indicating the potential use of these compounds for pharmaceutical applications.

## 2. Results and Discussion

### 2.1. Synthesis of Novel Thiophene Derivatives

**Scheme 1:**
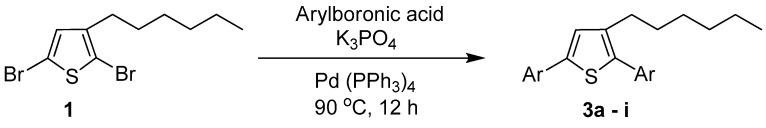
Synthesis of 2,5-biaryl-3-hexylthiophene (**3a**–**i**). **1** (1.0 mmol), Aryl-boronic acids (2.5 mmol), potassium phosphate (4.0 mmol), Pd(PPh_3_)_4_ (6 mol %), and H_2_O/Solvent (1:4), 90 °C, 12 h.

**Figure 1:**
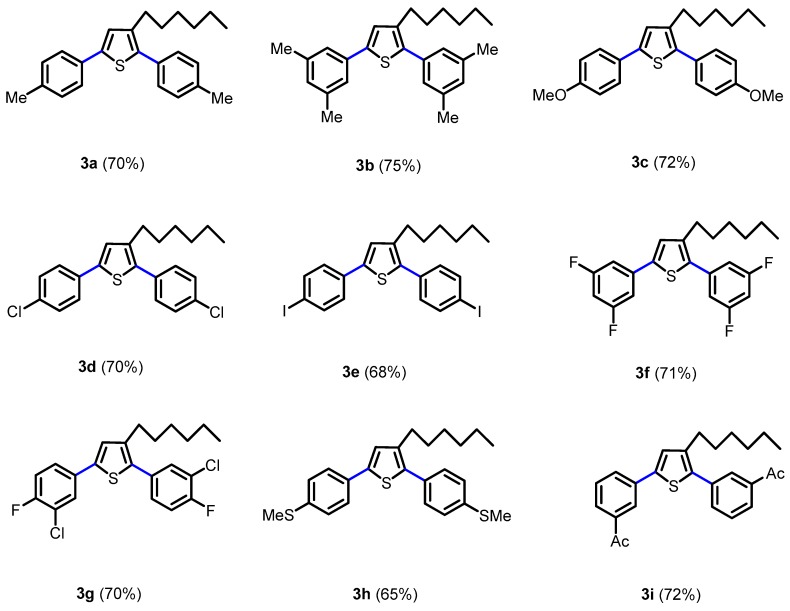
Synthesis of 2,5-biaryl-3-hexylthiophene derivatives (**3a**–**i**).

The synthesis of biaryls by the Suzuki coupling reaction of 4,5-dibromothiophene-2-carbaldehyde with several aryl-boronic acids has been reported elsewhere [17]. Similarly, more progress has been made exploiting the potential of using Suzuki coupling reactions, and several 4-arylthiophene-2-carbaldehyde derivatives have been reported in good yields [18]. Rasheed and coworkers [19] described the synthesis of 2-aryl-4-bromothiophenes and biaryl thiophenes from Suzuki cross-coupling reaction starting with several arylboronic acids as precursors. More recently, we have reported the synthesis of 5-aryl-2-bromo-3-hexylthiophene derivatives in moderate to good yields [10]. These thiophene derivatives were synthesized by using several aryl-boronic acids via Suzuki coupling reactions. The newly-synthesized thiophene derivatives were also explored for their biological application. Following our previous work, herein, we report the synthesis of some novel thiophene-based compounds by the application of Suzuki cross-coupling reactions [1]. To the best of our knowledge, the synthesis of 2,5-biaryl-3-hexylthiophene derivatives by using Suzuki cross-coupling reactions have not been explored to date. In brief, the present work describes the Suzuki reactions of 2,5-dibromo-3-hexylthiophene (**1**) with a number of aryl-boronic acids to produce corresponding biaryls. The optimal reaction conditions were achieved upon using potassium phosphate as a base, Pd(PPh_3_)_4_ as a catalyst, and temperature of 90 °C to obtain better yields. The Suzuki reactions of **1** (1 mmol) with several aryl-boronic acids (2.5 mmol) resulted in 2,5-biaryl-3-hexylthiophenes (**3a**–**i**) derivatives in a reasonable yield. The results revealed that the higher yields of these newly-synthesized thiophene derivatives were achieved, when 1,4-dioxane along with water used as solvent as compared to toluene. The higher yield of thiophenes in the case of former can be attributed to the high solubility of aryl-boronic acids in this solvent. It has been reported that the final yield of product depends on the type of solvent for synthesis [2]. The highest yield was obtained for solvent mixture at 1:4 (water/solvent), and is in accordance with the previous report for this solvent mixture [20]. In toluene, only moderate yields were attained due to less solubility of aryl-boronic acids in it. The results displayed in Scheme 1, Figure 1 reveals that the water/solvent ratio and temperature had a great influence of the final yield of the product during the synthesis of these novel thiophene derivatives.

### 2.2. Biological Applications

#### 2.2.1. Breast Cancer Cell Lines

Metastasis is the major reason of morbidity and mortality in cancer disease. In breast cancer patients, several tissues involving liver, lung, bone, and lymph node are affected by tumor involvement [21]. The anti-tumor activities of all newly-synthesized thiophene derivatives were screened for human breast cancer cell lines 4T1, MDA-MB-231, and MCF-7 using MTT (3-(4,5-dimethylthiazol-2-yl)-2,5-diphenyltetrazolium bromide) assay in vitro. The IC_50_ values for all the compounds are shown in Table 1. Cisplatin was used as a standard anti-tumor drug in this assay. The anti-tumor activity of these compounds against 4T1 cells revealed that, almost all the new compounds showed promising results. Compound **3h** showed the highest activity against 4T1 cells with IC_50_ value of 16 μM, which was better than the standard drug Cisplatin. The highest activity of **3h** might have been due to the presence of electron-donating methylthio group (-SMe) on aromatic phenyl ring. The compounds **3e**, **3g**, and **3d** exhibited good anti-tumor effects on 4T1 breast cancer cell line with IC_50_ values of 21.5 μM, 22 μM, and 22.8 μM, respectively. Similarly, the results revealed that the compounds **3a**–**c** exhibited moderate activity with IC_50_ values of 30.5 μM, 30 μM, and 29.5 μM, respectively. Moreover, the compound **3f** with electron-withdrawing fluoro groups (-F) was found to be the least potent against 4T1 cells with IC_50_ values of 31.5 μM.

The anti-tumor activity of all newly-synthesized compounds was also examined against MDA-MB-231 breast cancer cells. The results revealed that the compound **3e** exhibited highest anti-tumor activity with an IC_50_ value of 22.9 μM, which is much better than the reference drug Cisplatin with an IC_50_ value of 29 μM. It is likely possible that the electron-withdrawing iodo groups (-I) attached with phenyl ring might have been affecting the cytotoxicity of MDA-MB-231 cells. In contrast, the compound **3c** displayed lowest activity against MDA-MB-231 cells with IC_50_ value of 40 μM, which might be due to the effect of electron-donating methoxy group (-OMe) attached to this compound.

The anti-tumor activity of these compounds was also studied for MCF-7 breast cancer cell line. It was observed that almost all the compounds exhibited moderate to good activity against MCF-7 cells. The results revealed that the compounds **3a** was most active against MCF-7 cells with an IC_50_ value of 26.2 μM, displaying better results even than the positive control Cisplatin drug (IC_50_ value 48 μM). A likely reason for the high activity displayed by the compound **3a** might have its origin in the electron-donating effect of methyl groups on aryl ring. Surprisingly, the compound **3e** showed the lowest level of activity against MCF-7 cells with IC_50_ value of 91.4 μM.

#### 2.2.2. Anti-Thrombolytic Activity

**Figure 2:**
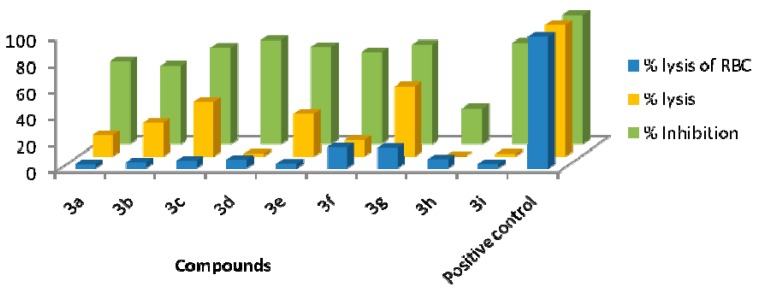
Anti-thrombolytic, biofilm inhibition, and haemolytic activities of 2,5-biaryl-3-hexyl thiophene-based compounds (**3a**–**i**).

Cerebral Venous Sinus Thrombosis (CVST) is a central nervous disorder, which is a common disease caused by thrombophilia and nephritic syndrome [22]. This fatal disease has been treated by the application of intravenous heparin and warfarin drugs [23]. Now a days, this disease is usually cured by thrombolytic drugs [24], such as urokinase and streptokinase, which are playing a significant effect in the cure of CVST [25]. In the present study, we have explored the potential of our newly synthesized thiophene derivatives for anti-thrombolytic activity by the procedure reported elsewhere [26]. The results revealed that the compounds **3g** and **3c** displayed the highest thrombolytic activity, while **3a**–**b** and **3e**–**f** exhibited reasonable activities when compared with the standard drug streptokinase. The compound **3g** exhibited the highest % lysis (53.12%) which, however, was still less than the standard streptokinase, which showed 100% lysis. This highest value of % lysis achieved by **3g** can be attributed to the electron-withdrawing fluorine and chlorine groups on the aromatic ring. In the same way, the compound **3c** also exhibited high value of % lysis (41.12%) compared to the positive control, which can be attributed to the -OCH_3_ groups on the ring. In contrast, Table 2 shows that the compounds **3d** and **3h**–**i** exhibited lowest hemolytic activity values. Furthermore, moderate hemolytic activity was shown by the compounds **3b** (25.32%) and **3e** (32.42%), which can be ascribed to the co-existence of methyl and bulky iodine groups on the phenyl ring. Overall, it can be seen that the thrombolytic activities displayed by the newly-synthesized compounds (**3a**–**i**) have a large influence coming from the type of functional groups and their position on the aryl ring (Table 2, Figure 2).

#### 2.2.3. Biofilm Inhibition Activity

Pathogenic micro-organisms form a biofilm, which mainly causes morbidness and mortality. This biofilm consists of conglomerations of bacterial cells and secured by a polymeric substance created by itself. Several fatal infections are caused by biofilms and they are difficult to remove [27]. The inhibition activities of all newly-synthesized thiophene derivatives (**3a**–**i**) against biofilm formation were assayed using the previously-reported method [28]. The experimentally-determined biofilm inhibition values for all compounds are expressed in Table 3. A selected strain of Gram-negative bacteria (*Escherichia coli)* was used to determine the anti-bacterial activity of all new derivatives at concentration of 50 μM/mL. The results revealed that the compounds **3c**–**e**, **3g** and **3i** expressed higher activity against *E. coli* showing values 73.27%, 78.85%, 73.80%, 75.53%, and 76.86%, respectively (Table 3, Figure 2). The compounds **3a,b**,**f**, however, showed moderate anti-microbial activity against the micro-organisms, when compared to the standard rifampicin. The lowest action against *E. coli* was shown by **3h** with a % inhibition value of 27.12%, which basically contain an electron-donating methylthio group. On the other hand, the compound **3d** displayed excellent biofilm inhibition activity (78.85%) when compared with the standard rifampicin. A likely possible cause of this high activity can be attributed to the electron-withdrawing substituents attached with the aromatic phenyl ring of **3d**, which might have been playing a significant role for increasing the anti-bacterial activity of this compound against Gram-negative bacteria. We believe that our newly-developed thiophene-based compounds with high % inhibition values can be potentially used to control the formation of biofilms.

#### 2.2.4. Haemolytic Activity

The newly-synthesized molecules (**3a**–**i**) were also examined for their hemolytic activity, following the method reported by Powell and co-workers [29]. The standard drug Triton X-100 was employed as a reference in these measurements. The results revealed that the compounds **3f** and **3g** showed the highest % lysis of red blood cells (RBCs), while **3b**–**d** and **3h** exhibited moderate % lysis values when compared with Triton X-100. The compound **3f** revealed highest haemolytic activity (15.91%), probably because of the existence of electron withdrawing fluorine groups present on 3 and 5 position of phenyl ring. Table 4 shows that the molecules **3a** and **3i** displayed small % lysis values (3.10%) and (3.27%), respectively. The results revealed that the compound **3h** exhibited moderate % lysis of RBCs (7.01%), which might be due to the bulky methylthio (-SMe) groups present at para position of the phenyl ring. The observed different values of % lysis suggested that the haemolytic activity of these compounds has affected by the existence of different functional groups attached on the ring. Ding et al. [30] reported that chloro group-containing molecules display facile haemolytic activity as compared with those compounds having a methyl group substitution on the ring.

## 3. Experimental Section

### 3.1. Characterization Techniques

^1^H- and ^13^C-NMR experiments were performed on a Bruker Aspect AM-400 (Bruker, Billercia, MA, USA) using CDCl_3_ and CD_3_OD to observe the values at 400/100 MHz. The chemical shift and coupling constant were measured in δ ppm and Hertz (Hz), respectively. A Buchi B-540 (Buchi, New Castle, DE, USA) instrument was utilized to check the melting points of all products. For the present research work, all necessary reagents were purchased from Alfa-Aesar (Ward Hill, MA, USA) and Sigma-Aldrich (St. Louis, MO, USA). A JMS-HX-110 spectrometer (JEOL, Peabody, MA, USA) was used to obtain EI-MS values of newly-synthesized derivatives. Silica gel 70–230 mesh, as well as 230–400 mesh, was used in column chromatography. Merck 60 PF_254_ silica gel TLC cards (Merck, Kenilworth, NJ, USA) were used to monitor the reaction properly. The UV lamp with wavelength in the range of 254 to 365 nm was used to identify the new products.

### 3.2. General Procedure for the Synthesis of 2,5-Biaryl-3-hexylthiophene (***3a–i***)

The substrate 2,5-dibromo-3-hexylthiophene (1 mmol) was taken in Schlenk flask and 6 mol% tetrakis(triphenylphosphine)palladium(0) was added along with 1,4-Dioxane (2 mL) under argon atmosphere. The mixture in the flask was stirred for half an hour at 25 °C. After mixing, the aryl-boronic acids (2.5 mmol), K_3_PO_4_ (4 mmol) and water (0.5 mL) were added in the mixture under argon atmosphere. The reaction mixture was cooled to normal room temperature after stirring the solution for 12 h at 90 °C. Ethyl acetate was used to separate the organic layer and then dried over magnesium sulfate. The excess solvent was evaporated using a rotary evaporator. The desired products were obtained from the purification of the crude residue using ethyl acetate and *n*-hexane in the same ratio by column chromatography. Spectroscopic techniques were applied for the characterization of the final products [18].

### 3.3. Characterization Data

*2,5-Bis(4-methylphenyl)-3-hexylthiophene* (**3a**). Mp: 165–168 °C; IR (KBr) 3035, 2960, 2890, 1481, 1465, 755, 725 cm^−1^. ^1^H-NMR (CDCl_3_ + CD_3_OD): δ = 6.99 (d, *J* = 7.98 Hz, 4H-Ar), 6.98 (d, *J* = 6.13 Hz, 4H-Ar), 7.35 (s, 1H-Thio), 2.36 (s, 6H-2CH_3_), 2.58 (t, *J* = 8.1 Hz, 2H-CH_2_), 1.30 (m, 6H-CH_2_), 1.65 (m, 2H-CH_2_), 0.93 (t, *J* = 6 Hz, 3H-CH_3_). ^13^C-NMR (CDCl_3_ + CD3OD): δ =13.13, 21.37, 23.74, 28.69, 29.01, 31.83, 33.20, 125.98, 126.36, 129.60, 130.71, 131.77, 133.07, 138.16, 138.46. EIMS (*m/z* + ion mode): 348.3: [M − C_6_H_13_]^+^ = 262.1: [M − CH_3_]^+^ = 247.1: [M − Benzene]^+^ = 171.1: [M − Thiophene]^+^ = 90.99: [M − CH_3_]^+^ = 75.99. Anal. Calcd. for C_24_H_28_S (348.54): C, 82.70; H, 8.10. Found: C, 82.72; H, 8.14%.

*2,5-Bis(3,5-dimethylphenyl)-3-hexylthiophene* (**3b**). Mp: 169 °C; IR (KBr) 3031, 2957, 2892, 1478, 1461, 750, 728 cm^−1^. ^1^H-NMR (CDCl_3_ + CD_3_OD): δ = 6.99–6.85 (m, 6H-Ar), 7.13 (s, 1H-Thio), 2.35 (s, 12H-4CH_3_), 2.65 (t, *J* = 6.98 Hz, 2H-CH_2_), 1.31 (m, 6H-CH_2_), 1.25 (m, 2H-CH_2_), 1.01 (t, *J* = 6.2 Hz, 3H-CH_3_). ^13^C-NMR (CDCl_3_ + CD_3_OD): δ = 13.29, 21.97, 22.79, 28.61, 28.89, 31.85, 32.21, 126.54, 127.65, 130.87, 133.01, 133.53, 138.16, 138.76, 139.04. EIMS (*m/z* + ion mode): 376.6: [M − C_6_H_13_]^+^ = 291.6: [M − 2CH_3_]^+^ = 261.4: [M − Benzene]^+^ = 186.4: [M − Thio]^+^ = 105: [M − 2CH_3_]^+^ = 75.1. Anal. Calcd. for C_26_H_32_S (376.60): C, 82.92; H, 8.56. Found: C, 82.95; H, 8.59%.

*3-Hexyl-2,5-bis(4-methoxyphenyl)thiophene* (**3c**). Mp: 164–166 °C; IR (KBr) 3028, 2954, 2885, 1475, 1459, 1230, 761, 731 cm^−1^. ^1^H-NMR (CDCl_3_ + CD_3_OD): δ = 7.60 (d, *J* = 6.30 Hz, 4H-Ar), 7.15 (d, *J* = 7.45 Hz, 4H-Ar), 7.10 (s, 1H-Thio), 3.78 (s, 6H-2OCH_3_), 2.64 (t, *J* = 7.72 Hz, 2H-CH_2_), 1.30 (m, 6H-CH_2_), 1.26 (m, 2H-CH_2_), 0.87 (t, *J* = 6.05 Hz, 3H-CH_3_). ^13^C-NMR (CDCl_3_ + CD_3_OD): δ = 13.65, 23.67, 28.58, 29.05, 30.98, 33.01, 55.82, 114.79, 126.01, 126.39, 128.64, 133.09, 138.18, 138.53, 160.72. EIMS (*m/z* + ion mode): 380.54: [M − C_6_H_13_]^+^ = 296.01: [M − OCH_3_]^+^ = 264.54: [M − Benzene]^+^ = 188.54: [M − Thio]^+^ = 106.96: [M − OCH_3_]^+^ = 77.01. Anal. Calcd. for C_24_H_28_O_2_S (380.54): C, 75.75; H, 7.42. Found: C, 75.77; H, 7.40%.

*2,5-Bis(4-chlorophenyl)-3-hexylthiophene* (**3d**). Mp: 167 °C; IR (KBr) 3032, 2949, 1471, 1455, 759, 741, 724 cm^−1^. ^1^H-NMR (CDCl_3_ + CD_3_OD): δ = 7.55 (d, *J* = 7.19 Hz, 4H-Ar), 7.73 (d, *J* = 7.90 Hz, 4H-Ar), 7.21 (s, 1H-Thio), 2.60 (t, *J* = 7.68 Hz, 2H-CH_2_), 1.32 (m, 6H-CH_2_), 1.27 (m, 2H-CH_2_), 0.90 (t, *J* = 6.12 Hz, 3H-CH_3_). ^13^C-NMR (CDCl_3_ + CD_3_OD): δ = 13.98, 22.64, 28.67, 29.03, 31.75, 32.31, 126.49, 128.83, 129.47, 131.73, 132.99, 134.19, 138.15, 138.89. EIMS (*m/z* + ion mode): 389.40: [M − C_6_H_13_]^+^ = 304.30: [M − Cl]^+^ = 268.80: [M − Benzene]^+^ = 192.85: [M − Thio]^+^ = 112.05: [M − Cl]^+^ = 75.96. Anal. Calcd. for C_22_H_22_Cl_2_S (389.38): C, 67.86; H, 5.69. Found: C, 67.90; H, 5.65%.

*3-Hexyl-2,5-bis(4-iodophenyl)thiophene* (**3e**). Mp: 182 °C; IR (KBr) 3027, 2952, 1474, 1452, 763, 729, 510 cm^−1^. ^1^H-NMR (CDCl_3_ + CD_3_OD): δ = 7.95 (d, *J* = 6.35 Hz, 4H-Ar), 7.50 (d, *J* = 7.78 Hz, 4H-Ar), 7.13 (s, 1H-Thio), 2.63 (t, *J* = 7.58 Hz, 2H-CH_2_), 1.31 (m, 6H-CH_2_), 1.25 (m, 2H-CH_2_), 0.87 (t, *J* = 6.23 Hz, 3H-CH_3_). ^13^C-NMR (CDCl_3_ + CD_3_OD): δ = 13.91, 23.15, 28.63, 29.11, 30.92, 32.29, 95.04, 126.27, 129.10, 132.62, 133.27, 138.17, 138.40. EIMS (*m/z* + ion mode): 572.28: [M − C_6_H_13_]^+^ = 487.28: [M − I]^+^ = 360.38: [M − Benzene]^+^ = 284.38: [M − Thio]^+^ = 203.38: [M − I]^+^ = 76.48. Anal. Calcd. for C_22_H_22_I_2_S (572.28): C, 46.17; H, 3.87. Found: C, 46.20; H, 3.88%.

*2,5-Bis(3,5-difluorophenyl)-3-hexylthiophene* (**3f**). Mp: 169–171 °C; IR (KBr) 3030, 2956, 1476, 1457, 1041, 759, 724 cm^−1^. ^1^H-NMR (CDCl_3_ + CD_3_OD): δ = 7.39 (s, 4H-Ar), 6.70 (s, 2H-Ar), 7.30 (s, 1H-Thio), 2.65 (t, *J* = 7.65 Hz, 2H-CH_2_), 1.35 (m, 6H-CH_2_), 1.30 (m, 2H-CH_2_), 0.90 (t, *J* = 6.23 Hz, 3H-CH_3_). ^13^C-NMR (CDCl_3_ + CD_3_OD): δ = 14.33, 22.70, 28.59, 29.07, 31.81, 32.19, 104.78, 112.04, 126.37, 133.08, 136.86, 138.15, 138.83, 165.23. EIMS (*m/z* + ion mode): 392.45: [M − C_6_H_13_]^+^ = 307.45: [M − 2F]^+^ = 269.45: [M − Benzene]^+^ = 193.40: [M − Thio]^+^ = 112.40: [M − 2F]^+^ = 75.0. Anal. Calcd. for C_22_H_20_F_4_S (392.45): C, 67.33; H, 5.14. Found: C, 67.37; H, 5.17%.

*2,5-Bis(3-chloro-4-fluorophenyl)-3-hexylthiophene* (**3g**). Mp: 172 °C; IR (KBr) 3034, 2955, 1470, 1455, 1075, 765, 740, 721 cm^−1^. ^1^H-NMR (CDCl_3_ + CD_3_OD): δ = 7.90 (s, 2H-Ar), 7.70 (d, *J* = 7.17 Hz, 2H-Ar), 7.20 (d, *J* = 6.90 Hz, 2H-Ar), 7.10 (s, 1H-Thio), 2.64 (t, *J* = 7.68 Hz, 2H-CH_2_), 1.30 (m, 6H-CH_2_), 1.25 (m, 2H-CH_2_), 0.90 (t, *J* = 5.98 Hz, 3H-CH_3_). ^13^C-NMR (CDCl_3_ + CD_3_OD): δ = 13.97, 23.31, 28.62, 28.99, 31.86, 32.13, 117.47, 121.29, 126.41, 127.29, 129.06, 130.92, 133.13, 138.17, 138.63, 159.29. EIMS (*m/z* + ion mode): 425.36: [M − C_6_H_13_]^+^ = 340.36: [M − Cl]^+^ = 304.86: [M − F]^+^ = 285.86: [M − Benzene]^+^ = 210.80: [M − Thio]^+^ = 129.80: [M − Cl]^+^ = 94.36: [M − Cl]^+^ = 75.36. Anal. Calcd. for C_22_H_20_Cl_2_F_2_S (425.36): C, 62.12; H, 4.74. Found: C, 62.13; H, 4.79%.

*3-Hexyl-2,5-bis(4-(methylthio)phenyl)thiophene* (**3h**). Mp: 174 °C; IR (KBr) 3027, 2957, 2870, 1467, 1451, 765, 723 cm^−1^. ^1^H-NMR (CDCl_3_ + CD_3_OD): δ = 7.70 (d, *J* = 8.20 Hz, 4H-Ar), 7.35 (d, *J* = 6.28 Hz, 4H-Ar), 7.13 (s, 1H-Thio), 2.65 (s, 6H-2SCH_3_), 2.60 (t, *J* = 7.63 Hz, 2H-CH_2_), 1.34 (m, 6H-CH_2_), 1.27 (m, 2H-CH_2_), 0.90 (t, *J* = 6.18 Hz, 3H-CH_3_). ^13^C-NMR (CDCl_3_ + CD_3_OD): δ = 14.04, 14.73, 22.79, 28.52, 29.12, 31.76, 32.13, 126.25, 127.37, 127.84, 130.20, 133.21, 138.02, 138.67, 139.69. EIMS (*m/z* + ion mode): 412.67: [M − C_6_H_13_]^+^ = 327.67: [M − SCH_3_]^+^ = 280.58: [M − Benzene]^+^ = 204.57: [M − Thio]^+^ = 123.57: [M − SCH_3_]^+^ = 75.95. Anal. Calcd. for C_24_H_28_S_3_ (412.67): C, 69.85; H, 6.84. Found: C, 69.85; H, 6.84%.

*2,5-Bis(3-acetylphenyl)-3-hexylthiophene* (**3i**). Mp: 181 °C; IR (KBr) 3025, 2962, 1690, 1469, 1453, 763, 731 cm^−1^. ^1^H-NMR (CDCl_3_ + CD_3_OD): δ = 7.95 (d, *J* = 8.15 Hz, 2H-Ar), 7.65 (t, *J* = 7.83 Hz, 2H-Ar), 8.05 (d, *J* = 6.35 Hz, 2H-Ar), 8.55 (s, 2H-Ar), 7.13 (s, 1H-Thio), 2.60 (t, *J* = 7.62 Hz, 2H-CH_2_), 1.33 (m, 6H-CH_2_), 1.28 (m, 2H-CH_2_), 0.90 (t, *J* = 6.05 Hz, 3H-CH_3_). ^13^C-NMR (CDCl_3_ + CD_3_OD): δ = 13.98, 23.19, 26.64, 27.89, 28.89, 30.89, 32.27, 126.03, 126.42, 128.87, 129.29, 130.96, 133.0, 133.67, 137.34, 138.18, 138.71, 197.01, 165.23. EIMS (*m/z* + ion mode): 404.56: [M − C_6_H_13_]^+^ = 319.56: [M − Ac]^+^ = 276.56: [M − Benzene]^+^ = 200.56: [M − Thio]^+^ = 119.56: [M − Ac]^+^ = 76.25. Anal. Calcd. for C_26_H_28_O_2_S (404.56): C, 77.19; H, 6.98. Found: C, 77.21; H, 6.95%.

### 3.4. Anti-Thrombolytic Activity

The anti-thrombolytic activity of all newly-synthesized thiophene derivatives was investigated by following the previously-reported method [26]. In a typical procedure, the sterile eppendorfs were filled with fresh venous blood taken from the human volunteers. The eppendorf tubes, which contained human blood (500 μL), were placed for 45 min at 37 °C to allow for clot formation. Once the clots were formed, the known volumes (100 μL) of sample solutions (**3a**–**i**) were added in eppendorfs having no serum in the blood samples. Basically, the serum was removed from blood prior to the clot formation stage. After the addition of samples, the tubes were incubated again for 90 min at 37 °C. For each experiment, the compound Streptokinase was used as a positive control, while water was used as a negative control. The anti-thrombolytic activity of newly-synthesized products was determined at percentage.

### 3.5. Biofilm Inhibition Assay

The newly-synthesized thiophene derivatives were assayed for their biofilm inhibition activity using the previously reported method [28]. For these measurements, the solutions of the thiophene derivatives were prepared in ethyl acetate. In brief, about 100 μL of sample solutions (**3a**–**i**) and 100 μL nutrient broth (Oxoid, Hampshire, UK) along with bacterial suspension (20 μL) were placed on the wells of 96-well sterile culture plates. Rifampicin was used as a positive control, while nutrient broth was used as a negative control for these biofilm inhibition activity measurements. The plates were covered and incubated for 24 h at 37 °C. Each plate was washed three times by using 220 μL of phosphate-buffered solution. After washing, each plate was shaken strongly in order to remove the non-adherent bacteria. 220 μL of 99% methanol solution was used to fix the remaining bacteria from these plates, followed by the drying in hot oven. After drying, the plates were stained with 220 mL of crystal violet with a concentration of 50% for five minutes. The excess stain from these plates was removed simply by using tap water. About 220 µL glacial acetic acid (33% concentration) was used to remove the dye attached with the cells. Each well was examined for OD value with the help of the micro-plate reader (BioTek, Winooski, VT, USA) at 630 nm.

### 3.6. Haemolytic Activity

The hemolytic activity of newly synthesized thiophene derivatives was examined by following the previously-reported method [29]. For these measurements, the solutions of the thiophene derivatives were prepared in ethyl acetate. In brief, about 3 mL of newly-obtained heparinized blood was taken in 15 mL sterile Falcon tube, which was later centrifuged for 5 min at 850 rpm. The supernatant was removed and washed three times and was later placed at room temperature for 30 min. In order to wash the thick pellet, 5 mL of ice-cold (4 °C) phosphate-buffered saline (PBS) having pH 7.4 was employed. The same PBS buffer (20 mL) was used to maintain the cells over longer periods of time. The erythrocytes present in cells were measured and diluted with PBS buffer. Approximately 180 µL of this suspension of erythrocytes was taken along with 20 µL of sample solution (**3a**–**i**) in eppendorfs. These eppendorfs were incubated thermally at human body temperature for 30 min, followed by centrifuging at 1310 rpm again for 5 min. After significant centrifugation, the supernatant was removed and diluted with phosphate-buffered saline (900 µL) and was later placed on ice. Each sample solution (200 µL) was kept in 96-well culture plates. For each measurement, phosphate-buffered saline and 0.1% Triton X-100 were employed as negative and positive control, respectively. A quant instrument was used to observe the absorbance of all sample solutions at 576 nm.

### 3.7. MTT Analysis

In brief, 0.8 × 10^5^ cells/mL were seeded in 96-well plates and were incubated in a humidified CO_2_ incubator overnight. The following day, serial dilution of the samples was performed with various concentration. The plates were left to incubate with the treatment for 72 h. After the designated time, 20 μL of MTT (5 mg/mL) was inserted into each of the wells and the cells were further incubated for an additional 4 h. Later, the media and the treatment were removed from all of the wells and 100 μL of DMSO was added. The absorbance plates were then read using a spectrophotometer (BioTek) at 570 nm.

## 4. Conclusions

The present study reports the synthesis of new 2,5-biaryl-3-hexylthiophene derivatives (**3a**–**i**) via the Suzuki-Miyaura cross-coupling reaction. The detailed investigations reveal that the nature of electron-donating and electron-withdrawing substituents present on the aromatic phenyl ring significantly influence the haemolytic, anti-thrombolytic, biofilm inhibition, and anti-tumor activities of these newly-synthesized thiophene derivatives. Several functional groups with different positions on phenyl ring were used to determine the efficiency and biological usefulness of various 2,5-biaryl-3-hexylthiophene derivatives (**3a**–**i**). The compounds containing electron-withdrawing groups on the ring showed good haemolytic, biofilm inhibition, and anti-thrombolytic activities. In particular, the compound 2,5-bis(3-chloro-4-fluorophenyl)-3-hexylthiophene (**3g**) displayed significantly high haemolytic and anti-thrombolytic activities, while the rest of derivatives exhibited mild to moderate activities. Furthermore, the compound 2,5-bis(4-chlorophenyl)-3-hexylthiophene (**3d**) displayed the highest biofilm inhibition activity against *E. coli.* Among the rest, 3-hexyl-2,5-bis(4(methylthio)phenyl)thiophene (**3h**) and 2,5-bis(4-methylphenyl)-3-hexylthiophene (**3a**) exhibited the best anti-tumor activity against 4T1 and MCF-7 cell lines with IC_50_ value of 16 μM and 26.2 μM, respectively.

## Figures and Tables

**Table 1:** Effect of 2,5-biaryl-3-hexylthiophene derivatives (**3a**–**i**) on the growth of three human cancer cell lines.

Entry	Compounds	IC50 (μM)		
		4T1	MDA-MB-231	MCF-7
1	**3a**	30.5 ± 0.08	36.8 ± 0.21	26.2 ± 0.70
2	**3b**	30.0 ± 0.81	32.2 ± 2.73	33.8 ± 0.29
3	**3c**	29.5 ± 4.30	40.0 ± 0.41	35.7 ± 0.09
4	**3d**	22.8 ± 0.04	24.8 ± 0.56	27.5 ± 0.67
5	**3e**	21.5 ± 3.71	22.9 ± 3.80	91.4 ± 0.59
6	**3f**	31.5 ± 1.10	28.1 ± 0.72	42.8 ± 2.87
7	**3g**	22.0 ± 0.57	26.2 ± 0.28	44.0 ± 0.17
8	**3h**	16.1 ± 0.09	29.2 ± 0.04	35.1 ± 3.8
9	**3i**	23.2 ± 0.08	36.8 ± 0.82	36.8 ± 0.03
10	**Cisplatin**	21.5	29.1	48.3

The results are average ± S.D of triplicate experiments, *p* < 0.05. Cisplatin was used as the control standard drug.

**Table 2:** The values of % lysis for newly-synthesized 2,5-biaryl-3-hexylthiophene derivatives (**3a**–**i**).

Entry	Compounds	% Lysis
1	**3a**	16.25 ± 0.028
2	**3b**	25.32 ± 0.092
3	**3c**	41.12 ± 0.035
4	**3d**	2.04 ± 0.091
5	**3e**	32.42 ± 0.046
6	**3f**	12.34 ± 0.073
7	**3g**	53.12 ± 0.063
8	**3h**	0.27 ± 0.021
9	**3i**	1.84 ± 0.083
10	**Positive control**	100

The results are average ± S.D of triplicate experiments, *p* < 0.05. Streptokinase was used as the control standard drug.

**Table 3:** Percent inhibition data of 2,5-biaryl-3-hexylthiophene derivatives (**3a**–**i**) at 50 μM/mL.

Entry	Compounds	% Inhibition
1	**3a**	62.50 ± 0.038
2	**3b**	59.48 ± 0.073
3	**3c**	73.27 ± 0.023
4	**3d**	78.85 ± 0.057
5	**3e**	73.80 ± 0.032
6	**3f**	69.68 ± 0.049
7	**3g**	75.53 ± 0.041
8	**3h**	27.12 ± 0.039
9	**3i**	76.86 ± 0.083
10	**Positive control**	97.43

The results are average ± S.D of triplicate experiments, *p* < 0.05. Rifampicin was used as a control standard drug.

**Table 4:** The % lysis of RBCs data for newly-synthesized 2,5-biaryl-3-hexylthiophene derivatives (**3a–i**).

Entry	Compounds	% Lysis of RBC
1	**3a**	3.10 ± 0.040
2	**3b**	4.54 ± 0.014
3	**3c**	5.80 ± 0.045
4	**3d**	6.66 ± 0.057
5	**3e**	3.62 ± 0.024
6	**3f**	15.91 ± 0.095
7	**3g**	15.57 ± 0.034
8	**3h**	7.01 ± 0.054
9	**3i**	3.27± 0.046
10	**Positive control**	100

The results are average ± S.D of triplicate experiments with *p* < 0.05. Triton X-100 was used as the control standard drug.
